# The Application of Gamma-Range Auditory Steady-State Responses in Animal Models: A Semi-Structured Literature Review

**DOI:** 10.3390/brainsci15111159

**Published:** 2025-10-28

**Authors:** Urte Jasinskyte, Cestmir Vejmola, Robertas Guzulaitis, Inga Griskova-Bulanova

**Affiliations:** 1 Institute of Bioscience, Life Sciences Center, Vilnius University, 10257 Vilnius, Lithuania; urte.jasinskyte@gmc.vu.lt (U.J.); robertas.guzulaitis@gmc.vu.lt (R.G.); 2 Psychedelic Research Center, National Institute of Mental Health, 250 67 Klecany, Czech Republic; cestmir.vejmola@nudz.cz; 3 Third Faculty of Medicine, Charles University, 100 00 Prague, Czech Republic; 4 Translational Health Research Institute, Faculty of Medicine, Vilnius University, 08406 Vilnius, Lithuania

**Keywords:** auditory steady-state responses, ASSRs, gamma, animal models, psychiatric disorders

## Abstract

**Background:** Gamma-range auditory steady-state responses (ASSRs) are emerging as promising translational biomarkers of neural network function. While extensively studied in human neuropsychiatric and neurodevelopmental research, their application in animal models has expanded in recent years, providing mechanistic insights into disease-related neural dynamics. However, methodological approaches vary widely, findings remain fragmented, and outcomes are not easily generalized. **Methods:** A literature search was conducted in March 2025 across PubMed and Scopus to identify studies investigating gamma-range ASSRs (30–100 Hz) in animal models with relevance to psychiatric and developmental conditions. **Results:** Most studies employed rodents, with a smaller number involving non-human primates, and used pharmacological, genetic, lesion-based, or developmental manipulations relevant to schizophrenia, autism spectrum disorder, and related conditions. ASSRs were highly sensitive to NMDA receptor antagonism, state- and trait-related factors, and exhibited region- and layer-specific generation patterns centered on the auditory cortex. Less common paradigms, such as chirps and gap-in-noise, also demonstrated translational potential. **Conclusions:** Animal research confirms that gamma-range ASSRs provide a sensitive, cross-species readout of circuit dysfunctions observed in psychiatric and neurodevelopmental disorders. To maximize their translational utility, future work should prioritize methodological harmonization, systematic inclusion of sex and behavioral state factors, and replication across laboratories. Strengthening these aspects will enhance the value of ASSRs as biomarkers for early detection, patient stratification, and treatment monitoring in clinical psychiatry.

## 1. Introduction

Gamma-range auditory steady-state responses (ASSRs) have been extensively studied in humans as potential neurophysiological markers for psychiatric and neurodevelopmental disorders [1]. These responses reflect neural entrainment elicited by periodic auditory stimulation, typically in the form of click trains or amplitude-modulated (AM) tones within the gamma frequency range (30–100 Hz) [2]. It should be noted, however, that synchronization within this range likely reflects contributions from distinct neural generators: lower gamma frequencies (30–50 Hz) are predominantly cortical, whereas higher frequencies (≥80 Hz) may involve more subcortical and brainstem sources, as the frequency range of phase-locked responses decreases along the ascending auditory pathway [3,4]. Because gamma ASSRs depend on the integrity of excitatory–inhibitory balance [5], gamma ASSRs are consistently altered in disorders such as schizophrenia (SZ), where deficits at 40 Hz have been reported in patients [6], in ultra-high-risk individuals [7], and even proposed as predictors of treatment outcomes [6,8]. Comparable alterations have also been described in autism spectrum disorders (ASD), where reductions in gamma synchrony are associated with impaired cortical network development [9]. While influential reviews such as O’Donnell et al. (2013) [1] established the promise of ASSRs as psychiatric biomarkers, they did not provide a structured synthesis of animal studies or systematically evaluate how different disease models reproduce alterations observed in patients. The present review addresses this gap.

Animal models allow controlled manipulation of neural circuits, neurotransmitter systems, and developmental trajectories, enabling mechanistic insights that cannot be achieved in humans. Moreover, the comparability of auditory stimulation paradigms across species makes ASSRs particularly suitable for translational research. Early experimental work in species such as cats [10], guinea pigs [11], gerbils [12], and rabbits [3] laid the foundation for understanding ASSR physiology, but recent efforts have shifted mainly to work on mice, rats, and monkeys to align more closely with translational psychiatry and disease modeling. Rodent models provide valuable mechanistic insight owing to their genetic manipulability and experimental accessibility, though their translatability to human neurophysiology remains limited across many domains [13,14,15,16]. Over the past decade, pharmacological [17,18,19], genetic [20,21,22], lesion-based [23,24], and developmental [25,26] models have been applied to study ASSRs, with the goal of reproducing patient-relevant alterations and probing underlying mechanisms.

Importantly, gamma ASSRs are not only descriptive readouts but also provide mechanistic information, as they index the capacity of neural circuits to sustain synchronized oscillations and reflect the balance of excitation and inhibition in cortical networks [5]. These mechanisms are directly implicated in psychiatric and neurodevelopmental disorders [25,27], where disrupted gamma synchrony has been linked to cognitive impairments in attention [28], working memory [29], and sensory processing [5]. Developmental animal models further allow the investigation of how ASSRs emerge and change across maturation, providing a window into altered trajectories relevant for ASD [30,31] and Fragile X syndrome (FXS) [25,32].

Despite their promise, findings from animal studies remain fragmented. Variability in stimulation protocols, recording methods, experimental states, and modeling strategies complicates cross-study synthesis and limits the integration of preclinical data into clinically meaningful frameworks. Addressing this challenge is essential, given the urgent need for objective biomarkers that can support early detection, patient stratification, and treatment monitoring in psychiatry. ASSRs are particularly promising in this regard because they are non-invasive, repeatable, and measurable across species using analogous paradigms.

Taken together, these considerations highlight the need for a structured synthesis of animal research on gamma-range ASSRs. This review addresses how gamma-range ASSRs are elicited, manipulated, and interpreted in animal models, and whether they reproduce alterations observed in patients. By focusing on disease-relevant models and excluding studies limited to basic auditory physiology without translational value, we aim to clarify the role of animal ASSRs as a cross-species biomarker, identify key methodological challenges, and outline future directions to strengthen their translational impact in developing clinically relevant biomarkers for diagnosis, prognosis, and treatment monitoring.

## 2. Materials and Methods

We conducted a semi-structured literature review to provide a focused overview of how gamma-range ASSRs are used in animal research. This approach enabled a systematic mapping of relevant studies while maintaining flexibility in selection, allowing us to emphasize works with translational relevance without being limited by overly rigid criteria. Unlike fully systematic reviews, our goal was not exhaustive coverage but a representative synthesis of current research.

To identify relevant studies, we conducted database searches in PubMed and Scopus (March 2025) using combinations of keywords related to auditory steady-state responses, animal models, and electrophysiological methods. Search strings included terms such as *“auditory steady-state response” OR “ASSR” OR “steady-state auditory evoked potential”* combined with *“animal model,” “rat,” “mouse,” “rodent,” or “monkey”* and with *“EEG,” “electrophysiology,” “evoked potential,” or “local field potential”.* The full keyword lists are provided in Appendix A.

The selection process followed predefined inclusion and exclusion criteria to ensure relevance to the study’s aim. We focused on original research articles presenting empirical findings; studies conducted in rodent models with additional inclusion of nonhuman primate studies relevant to neuropsychiatric disorders; research specifically assessing gamma-range ASSRs (30–100 Hz); studies utilizing electroencephalography (EEG), electrocorticography (ECoG), or local field potentials (LFPs) as core measurement techniques; and articles published in English. Only peer-reviewed publications were considered; preprints, unpublished data, and conference abstracts were excluded. We did not include non-original research (e.g., reviews, theoretical papers, meta-analyses, case reports); studies not published in English; research focused primarily on hearing mechanisms rather than on neurophysiological or neuropsychiatric implications of ASSR; and studies utilizing irrelevant auditory paradigms, such as transient evoked potentials instead of steady-state responses. The study selection process was independently conducted by two researchers, who screened article titles, abstracts, and full texts where necessary to ensure relevance. In cases where the abstract did not provide sufficient information, the full text was reviewed to confirm eligibility. Discrepancies between reviewers were resolved through discussion and consensus.

Because our aim was not the exhaustive coverage of all animal ASSR research but rather a synthesis with translational relevance, we applied an additional filter beyond database-based inclusion criteria. Specifically, studies were retained only if their design, outcomes, or manipulations allowed meaningful comparison to human neuropsychiatric or neurophysiological research. For example, reports focusing exclusively on auditory physiology (e.g., cochlear or brainstem responses without cortical readouts), or those limited to methodological validation without psychiatric or network-level interpretation, were excluded. Similarly, works using transient auditory evoked potentials rather than steady-state paradigms were not considered. This approach was chosen to maintain focus on findings with clear cross-species value for understanding psychiatric disorders, while acknowledging that it necessarily omits some technically relevant but less translationally oriented studies.

## 3. Results

**Figure 1:**
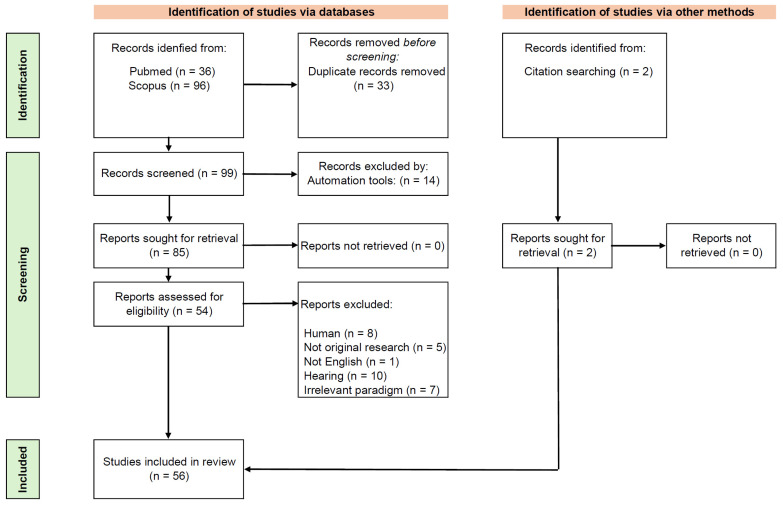
Scheme of studies inclusion process. **Top-left**: The records were identified in PubMed and Scopus databases. The duplicates were removed before screening. **Top-right**: The records identified from citation searching. **Middle**: The records were screened and excluded by predefined exclusion criteria. **Bottom**: 56 studies were included in the review.

The initial search identified 132 records (PubMed: 36; Scopus: 96) (Figure 1). After removing duplicates (*n* = 32), 100 unique articles remained (Figure 1). Following screening and the application of predefined inclusion criteria, 56 studies were included in this review (Figure 1, Table S1).

**Figure 2:**
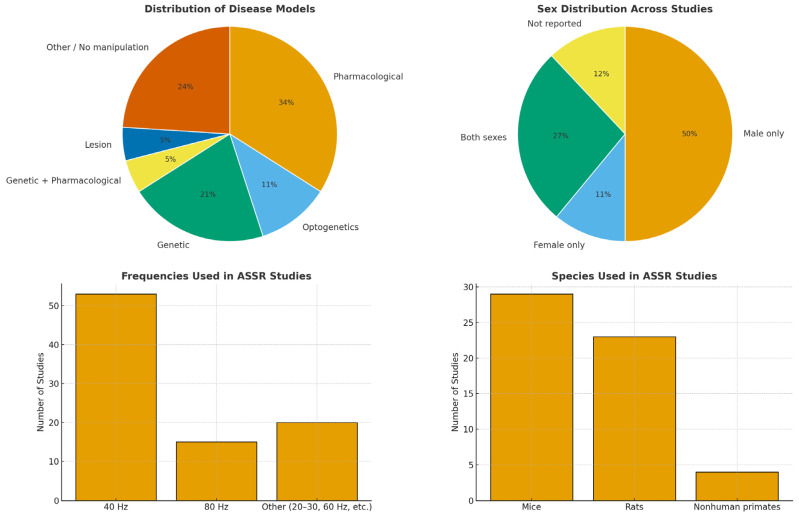
Summary of gamma-range ASSR animal studies included in the review. **Top-left**: Distribution of model types, showing the proportion of pharmacological, optogenetic, genetic, combined genetic-pharmacological, lesion-based, and other/no-manipulation approaches. **Top-right**: Sex distribution, showing the predominance of male-only studies and limited inclusion of females. **Bottom-left**: Stimulation frequencies used, with 40 Hz as the most common, followed by 80 Hz and other gamma-band ranges. **Bottom-right**: Species distribution, highlighting mice and rats as the most frequent models, with fewer studies in nonhuman primates.

Given the semi-structured nature of the review, some relevant studies may have been missed due to database limitations, keyword filtering, or the exclusion of gray literature. Nonetheless, this review provides a comprehensive and representative synthesis of current ASSR research in animal models relevant to neuropsychiatric disorders. Figure 2 summarizes the main findings after generalizing the included works.

### 3.1. Disease Models

Most studies employed mice (29) and rats (23), emphasizing their central role in ASSR-related translational research. A smaller number used nonhuman primates, including macaques (2), common marmosets (2), and rhesus monkeys (1). Mouse models included both wild-type (WT) and genetically modified strains (e.g., SRKO, Fmr1 KO, GRIN2A KO) to model ASD (13 studies), while rat studies predominantly utilized pharmacological agents to model SZ (16 studies), with fewer studies addressing other psychiatric or neurological conditions. Among the pharmacological models, MK-801 was the most commonly used drug (9 studies), followed by ketamine (7) and phencyclidine (PCP) (2) (all being non-competitive antagonists of the N-methyl-D-aspartate (NMDA) receptor complex), indicating a research preference for NMDA receptor antagonists.

### 3.2. Group Characteristics and Experimental Contexts

Regarding experimental design, 28 studies used only male subjects, 6 studies used only females, 15 used both sexes, and sex was not reported in 7 studies (Figure 1). The number of animals per study ranged from 5 to 164 in rodent models, and from 2 to 5 in nonhuman primate studies. Three studies did not report animal numbers. Regarding experimental conditions, freely moving animals were used in 40 studies, head-fixed setups in 13, and anesthetized conditions in 3.

### 3.3. Stimulation Protocols

Stimulation protocols covered a wide frequency range (1–480 Hz), focusing strongly on the gamma band (30–80 Hz). Stimuli were primarily click trains; however, some studies also employed AM tones, chirps, or gap-in-noise paradigms. Studies often used multiple stimulation frequencies, where 40 Hz was the most frequently used stimulation frequency (53 studies), followed by 80 Hz (15 studies), reflecting their relevance for gamma-range ASSR investigations (Figure 1).

## 4. Discussion

This section synthesizes key methodological patterns and findings across the reviewed studies, emphasizing their implications for understanding and modeling neuropsychiatric disorders. We examine how neurochemical manipulations and disease-specific models have been used to probe the mechanisms underlying gamma-range ASSRs, explore the neural circuitry and spatial organization of ASSR generation, and consider how brain traits and states, and methodological settings shape ASSR outcomes. These aspects are critical for improving the translational value of animal models and advancing the utility of ASSRs as neurophysiological markers of psychiatric and neurodevelopmental conditions.

We first consider how different disease models (pharmacological, genetic, lesion-based) utilize gamma-range ASSRs to capture circuit dysfunctions relevant to psychiatric and neurodevelopmental disorders. Next, we examine the neural circuits that generate ASSRs, highlighting cortical and subcortical contributions and their implications for understanding disorder-related alterations. We then explore how transient brain states (e.g., arousal, anesthesia, attention) and stable biological traits (e.g., sex, hormonal status) shape ASSR outcomes and influence translational relevance. Finally, we discuss methodological factors, including stimulation paradigms, recording approaches, and experimental designs that affect comparability across studies and limit cross-species translation

### 4.1. Disease Modeling

A central advantage of animal research lies in the ability to establish models that reproduce core features of neuropsychiatric and neurodevelopmental disorders. Gamma-range ASSRs have proven particularly useful in this context, as they provide a sensitive readout for circuit-level dysfunctions that parallel those observed in patients. SZ, where alterations of ASSRs have been consistently observed in clinical populations [33], has been modeled most extensively, using multiple approaches that converge on impaired gamma synchronization. Pharmacological induction of NMDA receptor hypofunction is widely accepted as a rodent model of SZ-like pathophysiology and consistently produces reductions in 40 Hz ASSRs, albeit with some variability across dose, brain region, and protocol (e.g., [17,34,35,36]). Notably, pharmacological NMDA receptor blockade evokes similar decreases in healthy humans [37,38], underscoring the translational validity of this approach. Complementary non-pharmacological models such as the neonatal ventral hippocampal lesion (NVHL) have also demonstrated persistent reductions in gamma-range ASSRs [23,39], supporting their relevance for developmental aspects of SZ.

Genetic models further expand this repertoire, including PLC-β1 knock-out (KO) mice [40], Grin1 mutants with glycogen synthase kinase-3 beta (GSK3β) modulation [20,41], and serine racemase (SRKO) mice modeling D-serine metabolism disruption [42], again mimicking ASSR reductions observed in humans.

Beyond SZ, ASD has been investigated in relation to gamma-range ASSRs [9], particularly in Fmr1 and PTEN mutants, where reductions in phase-locking and altered developmental trajectories [31,43,44] mirror findings of altered responses in human cohorts. Several studies have also extended ASSR applications to other conditions: systemic lupus erythematosus (anti-P antibody model, [45]), FXS with potential pharmacological rescue using minocycline [44], and inflammation-related changes induced by interferon-alpha (IFN-α) treatment [46].

Taken together, these diverse models indicate that gamma-range ASSRs constitute a unifying translational tool for probing circuit-level dysfunctions across neuropsychiatric and neurodevelopmental disorders. Notably, many alterations observed in animal studies closely parallel those reported in patients, underscoring the validity of ASSRs as cross-species biomarkers that bridge mechanistic investigations in animal models with clinically relevant phenomena in humans.

### 4.2. Localization of ASSRs and Neural Circuitry

While disease models demonstrate that gamma-range ASSRs are sensitive to neuropsychiatric and developmental pathophysiology, understanding the precise neural circuits underlying these alterations requires localization studies that animal models are uniquely positioned to provide.

Across models, the auditory cortex (AC) emerges as the dominant generator of these responses. For instance, Li et al. (2018) [24] demonstrated that ASSR deficits in the NVHL model were localized specifically to the primary AC, with non-primary auditory regions remaining unaffected. Consistent with this, Gautam et al. (2024) [47] showed that gamma synchrony emerged rapidly in the AC, highlighting its role as the earliest cortical locus of entrainment.

The prefrontal cortex (PFC) appears to play a more variable role. While some studies reported robust gamma entrainment (e.g., [44,48]), others failed to detect clear responses (e.g., [49]), suggesting that PFC involvement may be weaker, more state-dependent, or reliant on cross-regional interactions. Further structural specificity has been revealed by laminar recordings. Li et al. (2021) [50] found that 40 Hz responses were present across all cortical layers of the AC, with the strongest activity in the thalamorecipient granular layer. Johnson et al. (2024) [51] emphasized that superficial and deep layers are critical for temporal precision, supporting coordinated entrainment across networks. Optogenetic activation studies add to this picture: stimulating basal forebrain parvalbumin-positive neurons was shown to enhance AC–PFC coupling [52,53], providing causal evidence for top-down modulation in sustaining gamma synchrony [54,55].

In addition to cortical regions, subcortical structures also contribute to ASSR generation. The hippocampus and amygdala have been implicated in supporting gamma synchronization [49], and the thalamus is particularly critical as a relay structure. Wang et al. (2020) showed that NMDA receptor blockade in the medial geniculate body (MGB) significantly suppressed 40 Hz ASSRs in the AC but not in the PFC, reinforcing its role as a key driver of auditory gamma entrainment [45,46,56]. Methodological advances are beginning to bridge these findings across species. For example, Jiricek et al. (2021) [57] used multichannel EEG and source localization in rats to identify generators not only in auditory but also in motor cortices, with motor activity interpreted as reflecting behavioral freezing in response to stimulation. This highlights the importance of monitoring behavioral state when interpreting ASSR dynamics. They also highlight the variability that may arise due to methodological differences, behavioral or brain state fluctuations, and intrinsic network properties that influence the capacity to sustain gamma synchrony.

These neuroanatomical insights are not only mechanistically informative but also align with the circuits implicated in neuropsychiatric and neurodevelopmental disorders [58,59,60]. By linking AC dysfunction with distributed cortical–subcortical networks, animal models provide a translational framework for understanding how gamma synchrony disruptions manifest in conditions such as SZ and ASD.

### 4.3. State and Trait Modulation of ASSRs

Gamma-range ASSRs are sensitive to both state-dependent and trait-related influences, including arousal [61], consciousness levels [62], and biological sex [63]. Animal models provide unique opportunities to examine how these factors modulate neural synchrony, offering insights that are difficult to obtain in human studies and directly relevant to the variability observed in psychiatric populations.

Most experiments have been conducted in freely moving animals, allowing for naturalistic behavior but introducing variability in motor activity and internal brain state. Behavior was rarely tracked systematically, leaving distinctions between spontaneous movement and arousal-related effects underexplored. Li et al. (2020) [64], for example, reported that movement did not affect late-latency ASSRs and only influenced early-latency components, yet the influence of spontaneous versus evoked arousal remains unclear. Head-fixed preparations offer tighter experimental control but raise concerns about stress and altered arousal states, while anesthetized designs provide stability at the cost of suppressing neural synchronization. Wang et al. (2018) [49] observed significant reductions in 40 Hz ASSR power and phase-locking under anesthesia, mirroring reductions seen in humans during decreased consciousness [62]. In the same study, a mild arousing stimulus (foot shock) enhanced 40 Hz ASSRs and strengthened functional connectivity from auditory to medial PFC, pointing to a circuit mechanism by which arousal boosts sensory processing. These findings resonate with clinical reports showing altered arousal regulation in SZ [65] and mood disorders [66], where state fluctuations may exacerbate gamma synchrony deficits [67]. Importantly, no study has systematically compared different experimental states under matched protocols, underscoring the need for standardized or at least well-reported conditions to improve comparability across studies.

Experimental manipulations provide further evidence of state-dependent modulation. Optogenetic activation of basal forebrain parvalbumin-positive neurons altered cortical gamma activity in a state-dependent fashion (Kim et al., 2015 [48]), likely by synchronizing inhibitory networks [40]. Hwang et al. (2019) [52] showed that synchronized stimulation of these neurons enhanced ASSR magnitude and reorganized cortical topography in a manner consistent with increased attention, a finding directly relevant to attention deficits in SZ and ADHD [68]. In line, McNally et al. (2020) [69] reported that disruption of the ascending arousal system suppressed gamma ASSRs, resembling functional impairments observed in SZ [70]. Taken together, these findings suggest that arousal-related circuits exert powerful control over cortical gamma synchrony and provide mechanistic links to psychiatric symptom domains.

In addition to dynamic states, stable biological traits also influence ASSRs. Most reviewed studies used only male animals, limiting the understanding of sex-related differences. However, studies that included both sexes reported differences in amplitude, phase-locking, and developmental trajectories, particularly in models of FXS (Fmr1) and ASD (PTEN mutants) [31,43]. Male model mice showed more persistent deficits in 40 Hz phase-locking, whereas female mice exhibited earlier maturation of temporal processing. One study further suggested that the estrus cycle stage may modulate cortical oscillations [71], a finding consistent with prior human evidence [72]. Such sex and hormonal influences are clinically relevant, as women and men show different prevalence rates and symptom profiles in disorders such as SZ [73], ASD [74], and depression [75,76]. Furthermore, hormonal fluctuations are increasingly recognized as modulators of psychiatric symptom expression [77,78].

Taken together, evidence from animal models shows that both transient brain states (e.g., arousal, anesthesia, attention) and intrinsic traits (e.g., sex, hormone status) substantially shape ASSR dynamics. These influences parallel patient observations, where altered arousal regulation, attention deficits, and sex-related variability are hallmarks of neuropsychiatric and neurodevelopmental conditions. In addition, sample sizes varied widely, from very small groups of two to five nonhuman primates to over one hundred rodents, raising concerns about statistical power and reproducibility in the smaller studies. Accounting for brain states and ensuring adequately powered designs are essential for both experimental planning and data interpretation; these are prerequisites for establishing the translational validity of animal ASSR models.

### 4.4. Evidence from Non-Human Primates

Although rodent models yield important mechanistic insights, their relevance to human neurophysiology is often restricted. Research in non-human primates provides an essential translational link between rodent and human studies [79,80] of auditory steady-state responses. Although relatively few, these studies demonstrate that gamma-range ASSRs are conserved across primate species, with broadly comparable topography and frequency tuning to those observed in humans [81,82,83]. Moreover, pharmacological manipulations [83] and intracranial recordings [84] in awake animals confirm that cortical network entrainment can be reliably assessed in these models using EEG and ECoG techniques. Taken together, these findings highlight the translational value of primate models for validating neural mechanisms underlying ASSR generation and for bridging preclinical and clinical research.

### 4.5. Methodological Settings

Based on the reviewed studies, there is a notable degree of methodological consistency in the use of stimulation (e.g., 40–50 Hz clicks) to elicit responses, reflecting its established relevance for probing cortical synchronization. However, some also included other frequencies (e.g., 20, 25, 30, 60, 80 Hz), which may impact response profiles and limit direct comparability across studies. Importantly, less common paradigms such as chirps and gap-in-noise, though rarely used in human research, also demonstrated translational validity in animal models. All studies targeted auditory and frontal cortical areas, yet there was considerable variability in electrode placement (spanning primary auditory, frontal, and parietal regions) and in recording techniques (ranging from surface EEG, ECoG to LFP). These methodological differences may influence signal characteristics and comparability, and should be considered when interpreting findings, particularly in cross-study comparisons of signal strength, localization, and spectral characteristics.

From a translational perspective, reliance on a limited set of paradigms (primarily 40 Hz clicks) has the advantage of aligning with clinical protocols used in SZ and ASD research. However, expanding to more diverse paradigms may help uncover additional circuit properties relevant to disorders characterized by abnormal sensory processing, such as Fragile X syndrome or attention-deficit conditions. Likewise, greater methodological standardization, particularly in electrode placement, behavioral state tracking, and reporting practices, would improve reproducibility and enable clearer mapping between animal and human findings.

### 4.6. Limitation

It should be noted that our semi-structured approach deliberately prioritized studies with translational relevance to neuropsychiatric research, which means that some technically relevant but less clinically oriented reports (e.g., those focused solely on auditory physiology or methodological validation) were not included. While this narrows the scope, it strengthens the review’s focus on findings most directly comparable to human studies.

## 5. Conclusions

This review highlights the methodological diversity and translational potential of gamma-range ASSRs in animal models of neuropsychiatric and neurodevelopmental disorders. Across diverse models, ASSRs consistently emerged as a sensitive marker of cortical network function. In particular, their disruption in models of SZ and ASD supports their value for probing circuit-level dysfunctions that parallel human pathology. The use of pharmacological, genetic, lesion-based, and developmental models demonstrates that ASSRs can capture convergent deficits across distinct experimental approaches, reinforcing their potential as a cross-species biomarker. At the same time, methodological variability, including differences in stimulation paradigms, recording techniques, subject characteristics, and experimental states, remains a barrier to comparability and standardization. Taken together, animal ASSR studies reproduce key alterations consistently reported in SZ and ASD, while extending insights to other models (Fragile X, lupus, inflammation). Future research should prioritize methodological harmonization, systematic inclusion of female animals, and broader application of ASSRs across a wider range of disease models to strengthen their translational impact on understanding, diagnosing, and monitoring neuropsychiatric and neurodevelopmental disorders.

## Data Availability

Not applicable.

## Supplementary Materials

The following supporting information can be downloaded at https://www.mdpi.com/article/10.3390/brainsci15111159/s1. Table S1: Summary of studies included in the review. Refs. [50,85,86,87,88,89,90,91,92,93,94,95,96,97,98,99,100,101] are cited in Supplementary Materials file.

## Abbreviations

The following abbreviations are used in this manuscript:

AC	Auditory cortex
AM	Amplitude-modulated
ASD	Autism spectrum disorders
ASSRs	Auditory steady state responses
BF-PV	Basal forebrain parvalbumin
ECoG	Electrocorticography
EEG	Electroencephalography
EGF	Epidermal growth factor
FXS	Fragile X Syndrome
GABA	Gamma-aminobutyric acid
GlyT1	Glycine transporter 1
GSK3 β	Glycogen synthase kinase-3
IFN-α	Interferon-alpha
KO	Knock-out
LFP	Local field potentials
MGB	Medial geniculate body
NMDA	N-methyl D-aspartate
NVHL	Neonatal ventral hippocampal lesion
Pcdh10	Protocadherin 10
PCP	Phencyclidine
PFC	Prefrontal cortex
PV	Parvalbumin
SRKO	Serine racemase knock-out
SZ	Schizophrenia
TRN	Thalamic reticular nucleus
WT	Wild-type

## Appendix A

The keyword combination used for search in PubMed database is following: (“auditory steady-state response”[Title/Abstract] OR “ASSR”[Title/Abstract] OR “steady-state auditory evoked potential”[Title/Abstract]) AND (“animal model”[Title/Abstract] OR “rat”[Title/Abstract] OR “mouse”[Title/Abstract] OR “mice”[Title/Abstract] OR “rodent”[Title/Abstract] OR “monkey”[Title/Abstract]) AND (“EEG”[Title/Abstract] OR “electrophysiology”[Title/Abstract] OR “evoked potential”[Title/Abstract] OR “local field potential”[Title/Abstract]).

The keyword combinations used for search in Scopus database are following: “auditory steady-state response*” OR “ASSR” OR “steady-state auditory evoked potential*” OR “SSAEP” AND “animal model*” OR “rat” OR “mouse” OR “mice” OR “rodent*” OR “monkey” AND “EEG” OR “electrophysiology” OR “evoked potential*” OR “local field potential*” OR “LFP”.
