# Supplementary material for: The Application of Gamma-Range Auditory Steady-State Responses in Animal Models: A Semi-Structured Literature Review
Source: Brain Sci. 2025 Oct 28;15(11):1159. doi: 10.3390/brainsci15111159 (PMC12650100; doi:10.3390/brainsci15111159)
Supplement: Supplementary file 1 [file brainsci-15-01159-s001.zip › brainsci-3926548-supplementary.pdf]

brain sciences

MDPI

Table S1. Summary of studies included in the review.

Author & Year	Subjects / Animals	Sex / Experimental condition	Number of animals	Auditory Stimulation	Recording Technique & Location	Aim of Study	Main Results
Adraoui et al., 2024 [19]	Rats (PCP, MK-801, ketamine)	males, freely moving	12	50 Hz (clicks)	EEG, AC & frontal cortex	Investigate effects of aripiprazole on gamma oscillations in SZ model	Aripiprazole did not normalize MK-801-induced ASSR deficits
Aguilar et al., 2021 [85]	Mice (WT & SRKO)	males & females, freely moving	21	20, 30, 40, 50, 60 Hz (clicks)	EEG, frontal & parietal cortices	Characterize EEG biomarker deficits in NMDA receptor hypofunction SZ model	SRKO mice showed sensory gating impairments, but normal ASSR
Balla et al., 2020 [42]	Mice (WT & SRKO)	males & females, freely moving	100	20, 25, 40, 50, 80 Hz (clicks)	EEG, primary AC	Evaluate SRKO mice as a SZ model	SRKO mice showed increased ASSR power & phase locking, linked to parvalbumin (PV) interneuron dysfunction
Cizus et al., 2024 [86]	Mice (acute & chronic ketamine)	males & females, head-fixed	17 (10 m/7 f)	40 Hz (clicks)	ECoG, AC	Compare effects of acute vs. chronic NMDA suppression on brain oscillations	Acute NMDA suppression disrupted gamma oscillations, chronic suppression had less effect
Croom et al., 2023 [25]	Mice (WT & Fmr1 KO)	males, freely moving	62	40 Hz (Gap-in-noise paradigm)	Epidural EEG, AC & frontal cortex	Examine developmental trajectory of auditory temporal processing in ASD model	FXS model mice had delayed frontal cortex development affecting temporal processing
Croom et al., 2024a [43]	Mice (WT & PTEN conditional KO)	males & females, freely moving	145	40 Hz (Gap-in-noise paradigm)	Epidural EEG, AC & frontal cortex	Investigate developmental & sex differences in auditory	PTEN conditional KO mice showed hypersensitivity and

 https://doi.org/10.3390/xxxxx

						processing in ASD model	temporal processing deficits, worsening with age
Croom et al., 2024b [31]	Mice (WT & Fmr1 KO)	males & females, freely moving	115 (62 m/ 53 f)	40 Hz (Gap-in-noise paradigm)	Epidural EEG, AC & frontal cortex	Investigate sex differences in cortical temporal processing and ASSR in ASD model	Sex differences in Fmr1 KO mice affect ASSR & cortical temporal processing
Dejean et al., 2023 [87]	Mice (Shank3 mutation)	males & females, head-fixed	47 (21 m/ 26 f)	2-480 Hz (clicks)	Epidural EEG, AC & inferior colliculus	Develop method for detecting central auditory processing disorders in awake mice	Shank3 mutant mice showed impaired high-rate ASSRs
Gautam et al., 2023 [88]	Rats (MK-801)	females, freely moving	12	20, 40Hz (clicks)	Epidural EEG, PFC	Assess steady-state harmonic responses as pharmacodynamic biomarkers	Steady-state harmonic responses distinct from ASSR, MK801 affected phase synchrony differently
Gautam et al., 2024 [47]	Rats (MK-801)	females, freely moving	12	40 Hz (clicks)	Epidural EEG, AC & PFC	Compare local gamma synchrony in AC & PFC	MK801 enhanced synchrony in auditory cortex, disrupted it in PFC
Herzog et al., 2023 [22]	Mice (WT, GRIN2A & AKAP11 KO)	males & females, freely moving	71	10, 20, 30, 40, 50 Hz (clicks)	EEG, AC	Assess EEG biomarkers in SZ model	GRIN2A & AKAP11 KO mice had reduced 40 Hz ASSR and increased resting gamma power
Hwang et al., 2019 [52]	Mice (PV+ neurons)	males, freely moving	not reported	10, 20, 30, 40, 50 Hz (clicks)	EEG & optogenetics, AC & frontal cortex	Investigate role of basal forebrain parvalbumin (BF-PV) neurons in auditory cortical processing	Optogenetic BF-PV neuron stimulation enhanced 40 Hz phase locking & cortical topography

						and long-range cortical interactions	
Inaba et al., 2021 [89]	Rats (epidermal growth factor (EGF)-challenged)	males, freely moving	14–18	20, 40, 60, 80 Hz (clicks)	ECoG, AC	Assess auditory deficits following EGF perturbation in SZ model	EGF exposure reduced 40 Hz phase synchrony & spectral power in ASSR
Iwamura et al., 2022 [83]	Common marmosets (SKF-81297)	males & females, freely moving	5 (3 m/ 2 f)	20, 30, 40, 80 Hz (clicks)	EEG, scalp recordings	Study dopamine receptor modulation of ASSR in primates	Dopamine 1 receptor stimulation increased 40 Hz phase locking; Dopamine 2 receptor antagonism reduced it
Jasinskyte et al., 2023 [71]	Mice	females, head-fixed	20	10, 20, 40, 80 Hz (clicks)	ECoG, AC	Investigate ASSR reliability and changes during estrus cycle	40 Hz ASSR varied across estrus cycle, decreased in metestrus phase
Jiricek et al., 2021 [57]	Rats	males, freely moving	71	10, 43, 90 Hz (clicks)	EEG, cortical recordings	Evaluate feasibility of electrical source imaging in freely moving rats	12-electrode EEG system accurately mapped ASSR in rats
Johnson et al., 2024 [51]	Rats	males & females, freely moving	6 (3 m/3 f)	20, 30, 40, 50, 80 Hz (AM)	EEG & LFP, AC	Examine neural mechanisms underlying enhanced 40 Hz ASSR	40 Hz AM tones evoked strongest ASSR; deep layers of A1 coordinate response
Jonak et al., 2024 [26]	Mice (WT, Fmr1 KO)	males & females, freely moving	60	40, 80 Hz (click), 1–100 Hz range chirps	EEG, AC	Characterize developmental EEG biomarkers in Fmr1 KO mice	Fmr1 KO mice showed abnormal inter-trial phase coherence in 40 & 80 Hz ASSR
Kim et al., 2015 [48]	Mice (PV+ neurons)	not reported, head-fixed	8	40 Hz (clicks)	EEG & optogenetics, AC	Investigate role of BF-PV neurons in gamma oscillations	BF-PV neurons modulated cortical gamma power and

Konoike et al., 2022 [81]	Rhesus monkeys, marmosets	males & females, head-fixed	5 (2 m /3 f)	30, 40, 80 Hz (clicks)	Scalp EEG, comparative species study	Compare ASSRs across humans and non-human primates	ASSR Gamma-range ASSRs differed across species; marmosets & macaques had distinct optimal frequencies
Kozono et al., 2019 [90]	Rats (ketamine)	not reported, freely moving	16	10, 20, 30, 40, 50, 60, 70, 80 Hz (clicks)	ECoG, temporal and parietal cortices	Develop a standardized preclinical ASSR recording methodology	Temporal cortex recordings showed strongest ASSR, ketamine modulated response
Kozono et al., 2020 [21]	Rats (WT & Fmr1-KO rats)	males, freely moving	27	10, 20, 30, 40, 50, 60, 70, 80 Hz (clicks)	EEG, AC	Assess EEG biomarkers in SZ model	Fmr1-KO rats showed reduced in-ter-trial co-herence and event-related spectral pertur-bation at 40 Hz, linked to hyperactivity
Leishman et al., 2015 [91]	Rats (PCP)	males, freely moving	36	10, 20, 30, 40, 50, 55 Hz (clicks)	EEG, AC	Investigate acute vs. subchronic effects of PCP on auditory processing	PCP increased low-frequency ASSR but re-duced high-frequenc y response
Li et al., 2018 [24]	Rats (WT, NVHL)	males, head-fixed	12	40, 80 Hz (clicks)	LFP, primary & non-primary AC	Compare ASSRs between primary& non-primary AC in SZ model	NVHL impaired ASSR primarily in non-primary auditory cortex
Li et al., 2020 [64]	Mice	males, head-fixed	not re-ported	40 Hz (clicks)	LFP, AC, MGB, hippocampus, PFC	Assess effect of locomotion on ASSR	Locomotion reduced initial ASSR response but not late phase-locking
Li et al., 2021 [46]	Mice (IFN-α)	males, freely moving	10	40 Hz (clicks)	EEG, AC	Examine effects of IFN-α on ASSR and depres-	IFN-α reduced 40 Hz ASSR power, correlated with

						sive-like behavior	depressive-like behavior
Li et al., 2021 [50]	Mice	females, head-fixed	8	40 Hz (clicks)	LFP, AC	Determine laminar distribution of ASSR in AC	Strongest 40 Hz ASSR response recorded in granular layers of auditory cortex
Li et al., 2024 [92]	Mice	males, head-fixed	23	2, 4, 10, 20, 30, 40 Hz (clicks)	LFP & optogenetics, auditory thalamocortical pathway	Examine thalamocortical mechanisms of ASSR	Thalamic reticular nucleus (TRN) regulates 40 Hz ASSR via inhibition of MGB and cortical Gamma-aminobutyric acid (GABA)ergic interneurons
Lovelace et al., 2020 [44]	Mice (WT, Fmr1 KO, minocycline)	males, freely moving	24	40 Hz (clicks), 1-100 Hz range chirp	EEG, AC & frontal cortex	Assess minocycline effects on EEG abnormalities in ASD model	Minocycline treatment improved 40 Hz phase locking in Fmr1 KO mice
McNally et al., 2020 [69]	Mice (PV+ neurons)	males & females, freely moving	6	40 Hz (clicks)	EEG & optogenetics, cortical broadband recordings	Investigate role of BF-PV neurons on gamma oscillations & cognition	Optogenetic stimulation of BF-PV neurons modulated gamma power and cognition
Munch et al., 2023 [93]	Rats (ketamine)	males, freely moving	10	40 Hz (clicks)	LFP, AC	Investigate effect of ketamine on ASSR	Ketamine increased single-cell entrainment in auditory cortex during ASSR
Nakamura et al., 2022 [82]	Macaques	males, head-fixed	5	20, 40, 58.8, 83.3 Hz (clicks)	EEG, scalp recordings	Characterize ASSRs in macaques for translational research	Maximal ASSR in macaques at 83.3 Hz, unlike 40 Hz in humans
Nakao & Nakazawa, 2014 [20]	Mice (WT,	males, head-fixed	65	20, 40 Hz (clicks)	LFP, primary AC	Examine how NMDA receptor hypo-	40 Hz ASSR impaired in mutant mice,

	Ppp1r2-cre/fGluN1 KO)					function affects ASSRs & spontaneous LFPs in SZ model	with increased spontaneous LFP power
Nakao et al., 2020 [41]	Mice (Grin1 KO, GSK3 modulation)	males & females, head-fixed	10	40 Hz (clicks)	EEG, frontal cortex	Evaluate effects of GSK3 inhibition on gamma oscillations in SZ model	GSK3 β inhibition restored reduced 40 Hz ASSR and improved cognition
Port et al., 2017 [30]	Mice (WT, protocadherin10 (Pcdh10) KO, baclofen)	males & females, freely moving	37 (20 m/17 f)	10, 20, 40, 50, 60, 80 Hz (clicks)	ECoG, AC	Assess role of Pcdh10 in regulation of gamma oscillations & E/I balance in ASD model	Pcdh10 KO mice had reduced gamma-band ASSR; baclofen partially restored deficits
Prado-Gutierrez et al., 2019 [94]	Rats (anesthesia)	not reported, anesthetized	8	115 Hz (AM)	EEG, brainstem & AC	Assess improvements in ASSR detection through EEG epoch averaging	Independent EEG epoch averaging improved ASSR amplitude detection
Raza & Sivarao, 2021 [34]	Rats (MK-801)	females, freely moving	10	40 Hz (clicks)	Epidural EEG, AC	Assess test-retest reliability of ASSR & sensitivity to NMDA blockade	40 Hz ASSR showed superior test-retest reliability compared to tone-evoked gamma
Raza et al., 2023 [95]	Rats (clozapine, haloperidol)	females, freely moving	11	40 Hz (clicks)	EEG, PFC	Compare effects of clozapine & haloperidol on ASSR in SZ model	Clozapine enhanced ASSR phase resetting, haloperidol had limited effects
Rosenbrock et al., 2022 [18]	Mice (MK-801, iclepertin)	males, freely moving	21	40 Hz (clicks)	EEG, AC & PFC	Assess glycine transporter 1 (GlyT1) inhibition effects on ASSR deficits & cognition in SZ model	GlyT1 inhibitor reversed MK-801-induced ASSR deficits & improved memory
Schuelert et al., 2018 [17]	Mice	not reported,	24	40 Hz (clicks)	EEG, AC	Evaluate NMDA	NMDA antagonists

	(ketamine, MK-801)	freely moving				receptor antagonists as SZ model	impair EEG/event related potential responses, mimicking schizophrenia deficits
Shahriari et al., 2016 [40]	Mice (WT, PLC-b1 KO)	males, freely moving	13	20, 30, 40, 50 Hz (clicks)	LFP, AC & frontal cortex	Investigate auditory processing deficits in SZ model	ASSR power was reduced in AC and phase-locking was impaired
Sivarao et al., 2013 [35]	Rats (MK-801, nicotine, anesthetized)	males, anesthetized	not reported	10, 20, 40, 80 Hz (clicks)	EEG, AC	Investigate NMDA receptor modulation and nicotine effects on ASSR	MK-801 reduced 40 Hz ASSR power; nicotine reversed this effect
Sivarao et al., 2016 [36]	Rats (ketamine)	males, freely moving	12	40 Hz (clicks)	EEG, frontal cortex	Assess ASSR as a biomarker for cortical NMDA function	40 Hz ASSR is a biomarker for NMDA receptor function
Sullivan et al., 2015 [96]	Rats (MK-801, picrotoxin)	males, freely moving	18	10, 20, 40, 80 Hz (clicks)	LFP, primary AC	Examine NMDA & GABA-A receptor contributions to ASSR	Acute NMDA blockade increased ASSR coherence; chronic had no effect
Tao et al., 2025 [32]	Mice (WT & Fmr1 KO, NLX-101)	males & females, freely moving	164	40 Hz (Gap-in-noise)	Epidural EEG, AC & frontal cortices	Assess effect of serotonin receptor agonist on auditory processing in ASD model	NLX-101 improved auditory temporal processing in Fmr1 KO mice
Thankachan et al., 2019 [97]	Mice (TRN-PV+ neurons)	not reported, freely moving	5	40 Hz (clicks)	EEG & optogenetics, TRN & cortex	Determine role TRN PV neurons on EEG oscillations and ASSR in SZ model	Optogenetic inhibition of TRN PV neurons increased background delta & gamma power, impaired 40 Hz ASSR
Toader et al., 2020 [53]	Mice (PV+ neurons)	males, freely moving	15	40 Hz (clicks)	EEG & optogenetics, PFC	Study the role of PV interneurons	Optogenetic suppression of PV

						in PFC on ASSR and cognition	interneurons disrupted trial-to-trial reliability and increased oscillation power
Vohs et al., 2010 [23]	Rats (NVHL, muscimol, bicuculline)	not reported, freely moving	52	40 Hz (clicks)	EEG, AC	Study GABAergic modulation of ASSR in SZ model	GABA-A agonist modulated ASSR differently in schizophrenia model rats
Vohs et al., 2012 [39]	Rats (WT, NVHL, muscimol, ketamine)	males, freely moving	22	10, 20, 30, 40, 50 Hz (clicks)	LFP, AC	Examine NMDA & GABA contributions to ASSR in SZ model	NVHL & ketamine altered phase locking & power in 20-40 Hz range
Wang et al., 2018 [49]	Rats (anesthesia)	males, anesthetized	34	40 Hz (clicks)	LFP, AC, hippocampus, amygdala, PFC	Examine anaesthesia effects on ASSR and inhibitory gating	Chloral hydrate anaesthesia reduced ASSR power & phase locking
Wang et al., 2019 [98]	Rats	males, freely moving	14	40 Hz (clicks)	LFP, AC & medial PFC	Examine emotional modulation of ASSR	Emotional arousal increased 40 Hz ASSR power and coherence
Wang et al., 2020 [99]	Mice (MK-801)	not reported, freely moving	22	40 Hz (clicks)	EEG, auditory cortex & PFC	Investigate effects of NMDA blockade in MGB on ASSR	MK-801 in MGB suppressed 40 Hz ASSR
Wang et al., 2020 [45]	Mice (anti-P IgG, control IgG)	males, freely moving	6	40 Hz (clicks)	EEG, AC	Evaluate role of microglia in protecting against ASSR deficits in SLE model	Microglia protected against ASSR impairments induced by anti-P IgG
Yamazaki et al., 2020 [100]	Rats (Bicuculline)	males, freely moving	24	40 Hz (clicks)	EEG, AC	Investigate GABAergic modulation of ASSR	GABA-A antagonist reduced ASSR power and increased baseline gamma
Yan et al., 2023 [84]	Macaques	males, head-fixed	2	40 Hz (clicks)	ECoG, temporoparietal	Validate wireless BMI	Wireless BMI implant suc-

					cortex	implant for high-frequency EEG recording	cessfully recorded 40 Hz ASSR in macaques
Zhang et al., 2016 [101]	Rats (Saline, scopolamine, donezepil)	males, freely moving	19	10, 20, 40, 80 Hz (clicks)	LFP, AC	Study cholinergic modulation of ASSR	Scopolamine reduced 40 Hz ASSR, donepezil improved it
